# Fecal Bacterial Communities in treated HIV infected individuals on two antiretroviral regimens

**DOI:** 10.1038/srep43741

**Published:** 2017-03-06

**Authors:** Sandra Pinto-Cardoso, Catherine Lozupone, Olivia Briceño, Selma Alva-Hernández, Norma Téllez, Aguilar Adriana, Akio Murakami-Ogasawara, Gustavo Reyes-Terán

**Affiliations:** 1 Center for Research in Infectious Diseases, National Institute of Respiratory Diseases, Mexico City, Mexico; 2 Department of Medicine, University of Colorado, Denver, CO, USA

## Abstract

Intestinal microbiome changes that occur in HIV positive individuals on different antiretroviral therapy (ART) regimens are important to understand, as they are potentially linked with chronic inflammation and microbiome-linked comorbidities that occur at increased incidence in this population. We conducted a cross-sectional study comparing the fecal microbiomes of HIV-uninfected (HIV SN) to HIV-infected individuals on long-term ART (HIV+ LTART) from Mexico using 16S ribosomal RNA (16sRNA) targeted sequencing. These individuals were on two ART regimens based on either Non-Nucleoside Reverse Transcriptase Inhibitors (EFV) or ritonavir-boosted Protease Inhibitors (PI) with the same backbone of Nucleoside Reverse Transcriptase Inhibitors. Microbiome diversity was reduced in treated HIV infection compared to HIV SN (p < 0.05). Several operational taxonomic units (OTUs) related to the Ruminococcaceae family including *Faecalibacterium prausnitzii* were depleted in EFV and PI compared to HIV SN and negatively correlated with intestinal gut dysfunction as measured by the intestinal fatty binding protein (p < 0.05). This is the first report to address the fecal bacterial communities in HIV-infected individuals on two ARV regimens from Mexico.

Although the life expectancy of HIV-infected individuals has increased tremendously since the advent of combination antiretroviral therapy (ART), it still does not match that of HIV-uninfected individuals^1^. Indeed, non-AIDS noncommunicable diseases, such as cardiovascular disease^2^, neurocognitive^3^ and metabolic disorders remain a serious concern in treated HIV infection^4^. Interestingly, these noncommunicable diseases have all been linked with gut microbiome differences in non-AIDS populations^5^ suggesting that the gut microbiome may play an important role in the advent of non-AIDS complications that occur at increased incidence in HIV-infected individuals on ART^1^,^6^,^7^,^8^.

Emerging evidence suggests that bacterial communities found in the intestine (the microbiome) of HIV-infected individuals differ from those of HIV-uninfected individuals^9^,^10^,^11^,^12^,^13^,^14^,^15^,^16^,^17^. Several studies have shown enrichment in the bacterial genus Prevotella and a depletion of Bacteroides that occurs with untreated infection^9^,^17^,^18^ and persists with ART^18^ and others have also shown enrichment in Proteobacteria^11^ and reduced microbiota alpha diversity in HIV-positive individuals^10^,^13^. Alpha diversity has also been shown to be reduced with ART even compared to individuals with untreated infection^9^,^10^,^12^. However, high Prevotella and low Bacteroides were associated with men who have sex with men (MSM) behavior independent of HIV status^16^ indicating that many previous studies may have not used proper control populations for elucidating differences driven by HIV. A recent paper also showed enrichment for the *Prevotellaceae* family in the rectal mucosa of HIV-negative MSM engaging in condomless receptive anal intercourse (CRAI) compared to individuals who never engaged in anal intercourse^19^. Also relatively high Prevotella and Bacteroides have previously been described to be indicators of enterotypes found in the human gut of populations in Europe^20^,^21^ and the majority of individuals in both the US and Europe are dominated by Bacteroides-rich/Prevotella-poor microbiomes, whereas high Prevotella is common in agrarian cultures in the developing world^22^,^23^. However, previous studies have been mostly limited to populations in the U.S. and Europe, with relatively little attention given to sub-Saharan Africa^23^ and none to other parts of the world with high HIV rates. Thus, microbiome differences that occur with HIV still are not well understood overall.

Microbiome difference in HIV-infected individuals on ART may be driven by changes induced by HIV-infection itself that are un-resolved by ART or by the ART drugs themselves. Evidence for the latter is that ART regimens based on ritonavir-boosted Protease Inhibitors have been associated with increased incidence of non-infectious diarrhea^24^. ART-associated gastrointestinal symptoms, in particular non-infectious diarrhea^25^, together with unresolved HIV-associated gut mucosal dysfunction, have an impact on the quality of life^25^ and contribute towards treatment interruption or discontinuation^26^. Understanding microbiome differences that occur in the context of different drug classes may inform therapies aimed at manipulating the gut microbiome in treated HIV infection, which are still in their infancy^27^. To our knowledge, no studies have yet addressed directly the effect of different ART regimens on the gut microbiome composition and diversity. Therefore, we used 16S ribosomal RNA (rRNA) targeted sequencing to characterize the gut microbiome, via sampling the feces, of 33 HIV+ individuals on long-term suppressive ART on two different regimens: Efavirenz (EFV)-based regimen (n = 18) and ritonavir-boosted Protease-Inhibitors (PI)-based regimen (n = 15). We also investigated the impact of EFV- and PI-based regimens on markers of microbial translocation, gut epithelial barrier damage, and residual inflammation/immune activation. This is the first study to investigate the fecal microbiome of HIV-infected individuals in Mexico and one of the few to establish the baseline microbiome of HIV-uninfected individuals in Mexico (n = 10).

## Results

### Clinical characteristics of our cohort

Our cohort was composed of eight women (18.6%) and the mean age was 40.16 ± 10.14 years (19–66). Thirty subjects (69.77%) of our cohort lived in Mexico City, and the remaining 13 (30.23%) lived outside the capital, mainly in the state of Mexico. In the long-term ART (HIV+ LT ART) group, 20 (60.6%) were MSM, 10 (30.3%) were heterosexual (HTS) and 3 (9.1%) did not disclose their risk factor or the data was missing from their records. Data on sexual preference was not available for the control group. The controls were family members of the HIV-infected individuals. No differences in the body mass index (BMI; p = 0.7317), age (p = 0.6359), tobacco usage (p = 0.896), geographic location (p = 0.9397) and gender (p = 0.1377) were observed between HIV SN, HIV+ EFV and HIV+ PI. No differences in CD4 T cell count at the time of inclusion, duration of ART and BMI were observed between HIV+ EFV and HIV+ PI categories within the HIV-infected individuals on LT ART group. The demographic and clinical characteristics of our cohort are presented in Table 1.

### Bacterial diversity and communities' comparisons between HIV SN and HIV EFV and HIV PI

Across four different alpha diversity metrics, HIV-infected individuals on both EFV and PI regimens had significantly lower alpha diversity than HIV SN (Table 2; see Supplementary Figure S1 *for alpha diversity rarefaction curves*). There were no significant differences in alpha diversity between HIV+ individuals on EFV- versus PI-based regimens, indicating that long-term ART, irrespective of the regimen, is associated with overall lower bacterial diversity.

**Figure 1:**
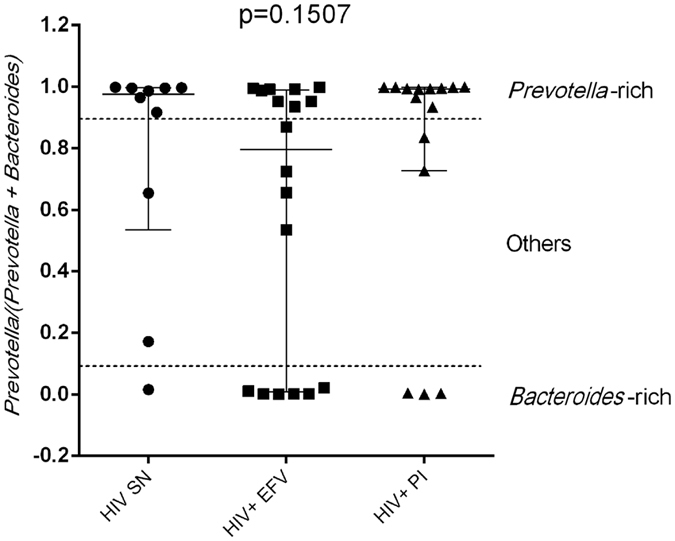
Scatter plot showing the *Prevotella* ratio in our cohort study. The *Prevotella* ratio was calculated as follows: the relative abundance of *Prevotella* was divided by the sum of the relative abundance of *Prevotella* and of *Bacteroides* as published in ref. 28. A value > 0.9 being *Prevotella*-rich and a value <0.1 being *Bacteroides* rich. Values in between >0.1- < 0.9 were considered as others. Data was plotted as the median with interquartile range. Non-parametric Kruskal-Wallis was used to compare the *Prevotella* ratio in our study groups using Graph Pad Prism version 6.

When looking at the bacterial communities in our study groups, we found a surprisingly high incidence of individuals in our HIV-SN group that had fecal microbiomes dominated by the genus *Prevotella* compared to *Bacteroides*. Indeed, we found a gradient of abundance between *Prevotella* and *Bacteroides*, with some individuals been *Prevotella*-dominant whilst others been *Bacteroides*-dominant as shown in Fig. 1. We defined *Prevotella* and *Bacteroides*-rich as described in ref. 28 and the *Prevotella* ratio was calculated as the relative abundance of *Prevotella* over the sum of the relative abundance of both *Prevotella* and *Bacteroides*, with a value >0.9 being *Prevotella*-rich and a value <0.1 being *Bacteroides* rich. Values in between >0.1- < 0.9 were considered as others. The fecal bacterial communities at genus level for our entire cohort are also shown in Supplementary Figure S2. High *Prevotella* in our HIV-SN control cohort (7 out of 10, 70%) is important to note because several previous papers have reported high *Prevotella*/low *Bacteroides* fecal microbiomes to be highly prevalent in HIV+ individuals, whether untreated or on ART 9,10. However, two more recent papers attributed these differences to sexual behavior^16^,^19^. Interestingly, the only other study describing the fecal microbiota of the Mexican population also found a dominance of the *Prevotella* genus in their control group (children aged 7–18 years^29^), indicating that *Prevotella*-rich community may be common in Mexican populations and might not be linked to sexual behavior.

**Figure 2:**
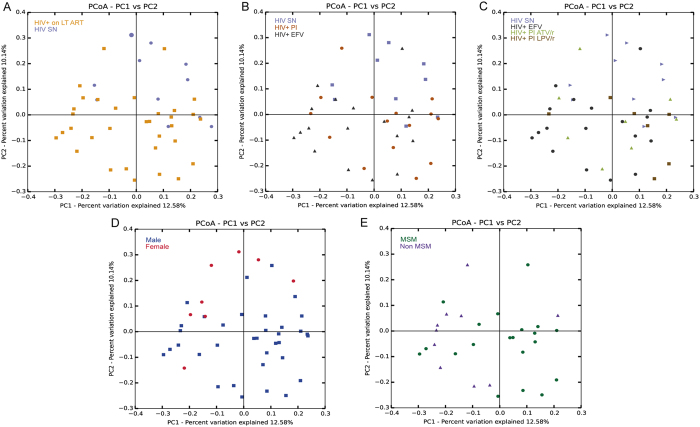
Unweighted UniFrac-based Principal component analysis (PCoA) showing the clustering of bacterial communities according to (**A**) HIV status, (**B**) HIV SN and ART regimens (**C**) HIV SN and the different subgroups of ART regimens, (**D**) gender and (**E**) sexual preference. Each dot represents a fecal sample. Two-D plots were generated from the unweighted_unifrac_Pc.txt file using the *make_2d_plots.py* script of QIIME. Abbreviations: HIV seronegative individuals (HIV SN), HIV-infected individuals on EFV-based regimen (HIV+ EFV) and HIV-infected individuals on PI-based regimen (HIV+ PI), ATV/r: Atazanavir/ritonavir, LPV/r: Lopinavir/ritonavir.

Next, we compared the overall microbial diversity between our groups using unweighted UniFrac distance matrices, which compares sample microbial diversity based on the degree of unique versus shared phylogenetic lineages that they contain^30^. Relationships were visualized with Principal Coordinates Analysis (PCoA; Fig. 2) and the Adonis statistical test for permutational multivariate analysis of variance was applied to the UniFrac distance matrix to test for significant associations between measured variables and microbiota composition. Samples clustered significantly by HIV status (Fig. 2A; Adonis R2 = 4.4%, p = 0.005). Adonis showed a larger R2 when the HIV+ group was further divided into different ARV regimens (EVF versus PI; R2 = 7.83%, p = 0.001, Fig. 2B) and when further subdivided into LPV and ATV (R2 = 10.37%, p = 0.001, Fig. 2C) indicating that differences between ART regimens are explaining some of the additional overall variation in community composition, despite no visual clustering by the ART regimens being apparent in the PCoA. No influence could be attributed to age, geographic location, BMI, or CD4 T cell count (p > 0.05). However, of the non-clinical variables tested, the samples did significantly cluster by gender (Fig. 1D; R2 = 0.0438; p = 0.001) and sexual preference (Fig. 1E; R2 = 0.167; p = 0.007). This result is in agreement with the recent report showing that fecal microbiomes are associated with MSM behavior independently of HIV^16^,^19^. Next, we tested which OTUs were discriminative between HIV SN and HIV+ on EFV versus PI regimens (see Supplementary Table S1). These included 2 OTUs related to *Faecalibacterium prausnitzii (F. prausnitzii;* Ruminococcaceae) and 2 OTUs related to the Ruminococcaceae family (Table S1). All 4 OTUs were significantly reduced in both HIV+ EFV and HIV+ PI in comparison to HIV SN (False Discovery rate (FDR) p < 0.05).

### Correlations between taxa and markers of disease progression and microbiome-linked comorbidities

**Figure 3:**
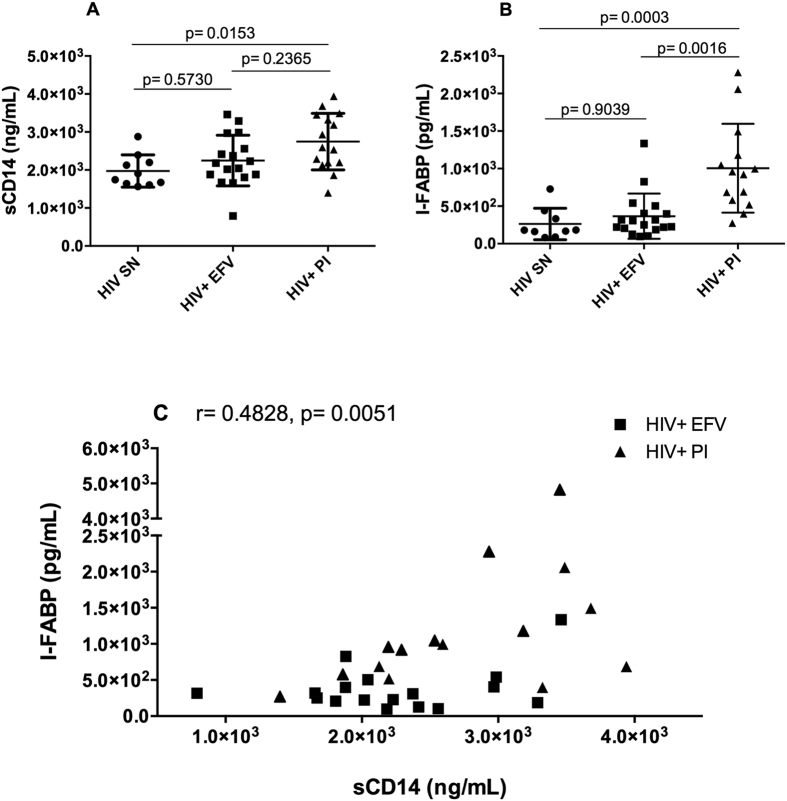
Scatter plots showing levels of plasma sCD14 (**A**) and plasma I-FABP (**B**) in our study groups. Data is plotted as mean ± standard deviation. Line represents the mean. Comparisons between groups were performed using nonparametric Kruskal-Wallis test. P values shown are adjusted for multiple comparisons. A significant correlation was found between sCD14 and I-FABP in the long-term ART group (**C**). Rho (r) and p-values refer to nonparametric Spearman’s correlation test.

First we addressed whether levels of microbial translocation and epithelial barrier damage were different in HIV+ EFV and HIV+ PI and also when compared to HIV SN by measuring plasma levels of sCD14 and I-FABP respectively. Soluble CD14 (sCD14) is released after monocyte activation in response to lipopolysaccharide^31^ and has been shown to independently predict mortality in HIV infection^32^. Intestinal fatty-acid binding protein (I-FABP) is present in epithelial cells of the mucosal layer of the small intestinal tissue and is released into circulation after mucosal tissue damage, and has been used as a biomarker of intestinal barrier dysfunction^33^. Plasma sCD14 levels were significantly higher in PI-based regimen when compared to EFV-based regimen (p = 0.0153) and comparable to EFV-based regimen instead of HIV + EFV (p = 0.2365) (Fig. 3A). We found significantly higher levels of plasma I-FABP in PI-based regimen compared to those on EFV-based regimen (p = 0.0016) and HIV SN (p = 0.0003) (Fig. 3B). Levels of plasma I-FABP and sCD14 in EFV-based regimen were comparable to HIV SN (p > 0.99 and p = 0.5730 respectively, Fig. 3A and B). As shown in Fig. 3C, we found a positive correlation between sCD14 and I-FABP in subjects on long-term ART (r = 0.4828, p = 0.0051). Taken together, these results seem to indicate that the ritonavir-boosted PI-based regimen is associated with mucosal barrier damage and bacterial translocation. We also investigated the impact of EFV- and PI-based regimens on markers of immune activation (as evaluated by the fraction of CD4+ and CD8+ expressing CD38 and HLA-DR) and inflammation (D-Dimer and high sensitivity C-reactive protein, hsCRP). Levels of T cell activation and inflammation markers did not significantly differ between the HIV-SN and HIV+ groups on the EFV and PI (see Supplementary Table S2).

Next, we further explored if any individual taxa correlated with markers of disease progression (sCD14, I-FABP, D-dimer, hsCRP, CD4+ and CD8+ T cell activation). We found 7 OTUs that negatively correlated with I-FABP (FDR p < 0.05) all belonging to the Clostridiales order (see Supplementary Table S3). This result is in agreement with our own results showing a lower abundance of the family Ruminococcaceae in both HIV+ EFV and HIV+ PI as compared to HIV SN. No correlation was found between individual taxa and sCD14 (FDR p > 0.98), CD4 T cell activation (FDR p > 0.93), CD8 T cell activation (FDR p > 0.99), D-dimer (FDR p > 0.96) and hsCRP (FDR p > 0.17).

**Figure 4:**
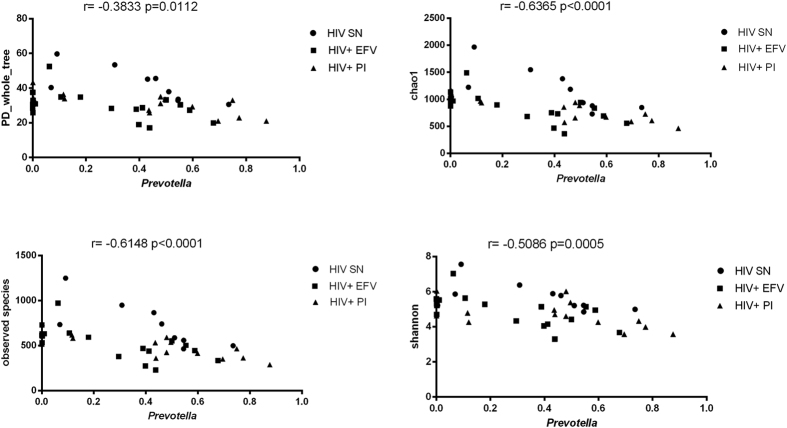
A negative correlation was found between the relative abundance of *Prevotella* and diverse indices of microbial diversity (PD-whole_tree, chao1, observed species and shannon). Rho (r) and p-values refer to nonparametric Spearman’s correlation test.

Because *Prevotella* and *Bacteroides* microbiomes have also been associated with certain diets high in carbohydrates/sugars and low in animal fat and protein in Western populations^21^, we explored whether the relative abundance of the genus *Prevotella* and *Bacteroides* was associated with dietary habits in our cohort. We found no associations between the relative abundance of *Prevotella* or *Bacteroides* and any dietary measurements including protein, carbohydrate, fiber, sugar, total fat, daily calorie and saturated fat intake (Supplementary Tables S4 and S5). We also explored correlations with indicators of obesity and found that individuals with *Prevotella* had higher Body Mass Index (BMI) compared to those with *Bacteroides* (p = 0.031, Supplementary Figure S4). Interestingly, as shown in Fig. 4, we found a strong negative correlation between the relative abundance of *Prevotella* and the four indices of microbial diversity, indicating that *Prevotella*-rich individuals have less microbial diversity than their *Bacteroides* counterpart in our cohort. Interestingly this result seems to be in disagreement with a previous study that reported higher alpha diversity in *Prevotella*-rich MSM versus *Bacteroides*-rich non-MSM individuals^16^.

## Discussion

In this study, we addressed the effects of antiretroviral drug classes on the gut microbial community composition of treated HIV infected individuals. We also compared them to non-HIV infected individuals in what is to our knowledge the first description of the fecal microbiome of HIV-infected individuals in Mexico. Interestingly, we found that 70% of our HIV-uninfected individuals (7/10) and 54.5% of our HIV treated individuals (18/33) had a fecal microbiota dominated by the genus *Prevotella* as defined in ref. 28. Only 1 HIV SN individual (10%) and 9 HIV treated individuals (27.2%) had a *Bacteroides*-rich fecal microbiota. This differs from what is typically reported in HIV-SN individuals in the US and Europe^22^,^23^. Interestingly, the only other study describing the fecal microbiota of the Mexican population also found a dominance of the *Prevotella* genus in their control group (children aged 7–18 years^29^), indicating that *Prevotella*-rich enterotypes may be common in Mexican populations. This result is unlikely attributed to diet as has been in other populations^21^ since Mexicans do not typically eat a particularly carbohydrate rich/fat/protein poor diet and indeed, we did not see any correlation between *Prevotella* and dietary measurements in our cohort. However, we did find a correlation between *Prevotella* and high BMI; *Prevotella* had previously been associated with obesity in one study^34^ but other studies have looked for and not found this association^35^.

HIV+ individuals on long term ART had significantly lower alpha diversity compared to HIV-SN individuals regardless of ART regimen. This result is consistent with that of multiple other studies that have shown decreased alpha diversity with HIV infection compared to SN, and with ART even compared to HIV-untreated^10^,^14^. Interestingly, HIV+ individuals on long term display lesser relative abundance of key players implicated in maintaining gut homeostasis including *F. prausnitzii* 36. Interestingly, decreased *F. prausnitzii* abundance has been linked to both gut dysbiosis and inflammatory bowel disease^37^,^38^. *F. prausnitzii* is usually found in abundance in the fecal microbiota of healthy individuals and is a source of energy for colonocytes through the production of butyrate from the fermentation of dietary fiber^39^. In addition, we found a negative correlation between I-FABP, a marker of intestinal dysfunction, and bacteria from the Clostridiales order including *F. prausnitzii* and Roseburia, both known butyrate producers^40^, further indicating the vital importance of these bacteria in maintaining a healthy gut homeostasis. When looking at others markers of disease progression, we found that HIV+ PI had significantly higher sCD14, a marker of bacterial translocation compared to HIV SN but not HIV+ EFV. We think that these results might be explained by the fact that non-infectious diarrhea is more often reported in ARV regimen based on PI^24^,^25^,^26^.

We are aware that our study has several limitations. First, we lack data on the sexual preference of our control group that is needed to assess the impact of HIV on the gut microbiome while controlling for sexual behavior. Interestingly, Prevotellaceae enrichment was found in most but not all MSM who engaged in CRAI^19^ suggesting that the factors driving *Prevotella*-rich microbiotas in MSM population are still unclear. In the light of recent publications^16^,^19^, we are actively recruiting HIV-negative MSM to characterize their fecal microbiome and continue our research goal, which is to truly understand changes in the gut microbiome that are driven by HIV. Also, the number of HIV SN individuals is relatively small and should be increased to fully characterize the baseline microbiome of HIV-uninfected individuals in Mexico. Also, we did not establish the bacterial fecal microbiome of HIV-infected individual’s naive to ART. Additionally, these results are largely based on descriptive observations from a cross-sectional cohort. Further investigation is required to determine their relevance. However, we believe that these findings are a further step towards elucidating the role the gut microbiome in non-communicable diseases and mucosal immune dysfunction of individuals who are infected with HIV and on ART.

In conclusion, we showed that the long-term ART treated individuals have lower bacterial diversity as compared to HIV SN individuals. At the taxonomic level, subtle differences were observed between treated HIV individuals on either Efavirenz or PI-based ARV regimens and HIV SN. However, with our current dataset, we can not conclude whether these differences are attributed to HIV infection or sexual behavior. Further work is needed to understand gut microbiota dynamics under ART by means of extensively characterized cohorts and longitudinal studies.

## Methods

### Ethics statement

This protocol was evaluated and approved by the Ethics Committee of the National Institute of Respiratory Diseases (Mexico City, Mexico). Our study was conducted according to the protocol guidelines of our Institution, and approved by the Ethics Committee of the National Institute of Respiratory Diseases (Mexico City, Mexico). All participants were adults (over 18 years old) and gave written informed consent in accordance with the Declaration of Helsinki. Samples from the 43 subjects enrolled in this study were collected and analyzed at the Center for Research in Infectious Diseases of the National Institute of Respiratory Diseases.

### Study population

Our cohort was composed of 43 adult subjects; 10 were HIV seronegative (HIV SN) and 33 were HIV-infected individuals (HIV+) on long-term ART. Long-term ART was defined as HIV-infected individuals having undetectable plasma viral loads for at least 2 years from the time of inclusion in this study. Our control group (HIV SN as determined by negative ELISA assay) was enrolled amongst the family members of our participating HIV-infected individuals. In the HIV+ cohort on long-term ART, 18 subjects were on an Efavirenz/Tenofovir Disoproxil Fumarate/Emtricitabine (EFV/TDF/FTC)-based therapy (HIV+ EFV) and 15 were taking ritonavir-boosted protease inhibitor-based therapy (HIV+ PI): Atazanavir/ritonavir (ATV/r, n = 8) or Lopinavir/ritonavir (LPV/r, n = 7) with a backbone of Tenofovir Disoproxil Fumarate/Emtricitabine. None of the HIV+ subjects on long-term ART presented with apparent active opportunist infections at the time of recruitment. Also, none of the subjects (HIV SN and HIV+ EFV/PI) were taking antibiotic treatment at the time of recruitment or within a month prior to sample collection. The mean time between sample collection and last antibiotic treatment self-reported by each subject was 7.5 ± SD 4.59 months (see Supplementary Table S6). Demographic data was surveyed via the use of a questionnaire at the time of recruitment. Dietary intake was assessed by CIENI´s nutritionist team by means of a food frequency questionnaire (FFQ) validated for the Mexican population and three 24-hour dietary recalls as described elsewhere^41^,^42^. Total body composition and fat content was measured via Dual-energy X-ray absorptiometry (DXA).

### Plasma viral load and CD4 measurements

HIV plasma viral load was determined by automated real time polymerase chain reaction (PCR) using the m2000 system (Abbott, Abbott Park, IL, USA) with a detection limit of 40 HIV RNA copies/mL. CD4+ T cell counts were obtained by flow cytometry using the Trucount Kit in FACSCanto II instruments (BD Biosciences, San Jose, CA).

### Collection of fecal samples and DNA extraction

To characterize the gut microbiome, fecal samples were collected by study participants in a sterile container provided by the CIENI-INER shortly before their clinic visit and stored at −80 °C until DNA extraction. DNA was extracted using the QIAamp DNA stool minikit (Qiagen Inc., Valencia, CA, USA) according to the manufacturer’s instructions. Bacterial DNA concentration was measured by fluorometry using the Qubit^®^ dsDNA high-sensitivity assay kit and the Qubit^®^ 2.0 fluorometer (Life Technologies, Carlsbad, CA, USA) as instructed by the manufacturer. DNA purity was also assessed by absorbance on a Nanodrop N1000 (Thermo Fisher Scientific, Carlsbad, CA, USA) by measuring the A260/A280 ratio. DNAs with an A260/A280 ratio of 1.8–2.0 were used for PCR amplification.

### 16S rRNA targeted sequencing

16S libraries were prepared as instructed in the 16S Metagenomic Sequencing Library Preparation (Illumina, San Diego, CA, USA). Primers to amplify the V3-V4 regions were previously published^43^. The library was denatured with 0.2N NaOH, combined with PhiX internal control (25%) and loaded on a MiSeq reagent kit V3 (2 × 300 cycles; Illumina). Primary analyses were performed on the MiSeq instrument. Paired end reads were joined using *join_paired_ends.py* of QIIME using the fastq_join method of QIIME (Quantitative Insights into Microbial Ecology^44^). Reads were quality filtered using the *split_library_fastq.py* script of QIIME. Sequences were then concatenated in a single file (seqs.fna) and further analyzed using QIIME version 1.9.0 on an Ubuntu machine.

### Analysis of V3-V4 16S amplicon data

To assign operational taxonomic units (OTUs) at 97% identity and perform taxonomic classification, we used UCLUST^45^ and the RDP Classifier^46^ via the QIIME *pick_closed_reference_otus.py* script and Greengenes version 13_8 reference database (http://greengenes.secondgenome.com/). The OTU table was rarified at a sequencing depth of 25,025 sequences per sample. A total of 4152 unique OTUs were found. Bacterial communities were summarized at various taxonomic levels using the *summarize_taxa_through_plots.py* script of QIIME. To estimate within sample microbiome diversity, four different metrics were calculated including: Chao1 (estimated OTU richness), observed_species (the number of species present in a given sample), Shannon (the number of species present in a given sample and their relative abundance) and PD_whole_tree (Faith´s phylogenetic diversity^47^). We also calculated Goods Coverage, and sample coverage was in the range of 0.988–0.992 suggesting that bacterial richness had been sampled adequately. Alpha diversity metrics were compared using the *compare_alpha_diversity.py* script using a non-parametric t-test at a deepest rarefaction depth. P values were FDR-corrected for multiple comparisons. Beta diversity (unweighted and weighted Unifrac^30^) was calculated using the script beta_diversity_through_plots.py script of QIIME at a sample depth of 25,025 sequences. Principal Coordinates analysis plots were generated for both unweighted and weighted Unifrac data to visualize the microbial communities based on the metadata categories provided in the mapping file, corresponding to our study cohort. We used the nonparametric Adonis method on both unweighted and weighted distance matrices obtained to determine which measured variables best explained the clustering of microbial communities by means of the *compare_categories.py* script of QIIME.

### Measurements of plasma soluble markers

Peripheral blood (36mls) was collected at the time of recruitment in EDTA-coated tubes. After centrifugation, plasma samples were immediately frozen at −80 °C until use. Enzyme-Linked Immunosorbent Assays (ELISA) were used to measure soluble CD14 (sCD14, R&D Quantikine^®^ DC140, R&D systems, Minneapolis, MN, USA), I-FABP (Intestinal Fatty Acid Binding Protein, HK406-02, Hycult Biotech, Uden, The Netherlands), hsCRP (High Sensitive C-Reactive Protein, HK369, Hycult biotech) and D-Dimer (RayBiotech, ELH-DDIMER, Norcross, GA, USA) according to the manufacturer’s instructions. Samples and standards provided in each kit were measured in duplicate. The average value was used as reference. ELISA plates were read in an ELx808 absorbance microplate reader (BioTek, Winooski, VT, USA).

### Measurement of CD4 and CD8 T cell activation

Activated CD4 and CD8 T cells were identified using the following staining panel: brilliant™ violet (BV) 570-conjugated anti-CD3 (clone: UCHT1), phycoerythrin (PE) Cyanine5.5-conjugated anti-CD4 (clone: MHCD0418), BV605-conjugated anti-CD8 (clone: RPA-T8), CD38 BV711 (clone: HIT2) and BV785-conjugated anti-HLADR (clone: UCHT1). All conjugated antibodies were purchased from Biolegend (San Diego, CA, USA) except for the PE-Cy5.5 conjugated anti-CD4, which was purchased from Invitrogen. CD4+ and CD8+ T cells were gated within single, viable CD3+ lymphocytes. Appropriate controls including fluorescence minus one controls, were done. Data analysis was performed with FlowJo V10 software (Tree Star, Ashland, OR, USA). Flow Staining was acquired within 24 hours of staining on a LSRII Flow (Becton Dickinson Biosciences, San Jose, CA, USA).

### Statistical analysis

Correlations were evaluated with the nonparametric Spearman test and comparisons between groups were done using Kruskal-Wallis test correcting for multiple corrections with Dunnett´s post-test unless stated otherwise. Graphs and tests were done using Graph Pad Prism version 6.01 (La Jolla, CA, USA). Correlations between taxa and with markers of disease progression were performed using Spearman’s Rho method (observation_metadata_correlation.py script of QIIME).

## Additional Information

**Accession Codes**: Raw 16s rRNA sequences were submitted to the Sequence Read Archive (SRA) under BioProject PRJNA344791.

**How to cite this article**: Pinto-Cardoso, S. *et al*. Fecal Bacterial Communities in treated HIV infected individuals on two antiretroviral regimens. *Sci. Rep.* **7**, 43741; doi: 10.1038/srep43741 (2017).

**Publisher's note:** Springer Nature remains neutral with regard to jurisdictional claims in published maps and institutional affiliations.

## Supplementary Material

Supplementary Information

## Figures and Tables

**Table 1:** Clinical and Demographic characteristics for our study population.

	HIV SN	HIV+ EFV	HIV+ PI	P-value
N	10	18	15	—
Age (Years)	39 ± 11.72 (20–66)	41 ± 8.93 (24–59)	39 ± 9.76 (19–54)	p = 0.6359
Sex (Female)	4/10 (40%)	2/18 (11.10%)	2/15 (13.33%)	p = 0.1377
**Geographic location**				
Mexico City	8/10	11/18	11/15	p = 0.9397
Others	2/10	7/18	4/15	
**Risk group**				
MSM	10/10	12/18	8/15	—
HTS		5/18	5/15	
Data missing		1/18	2/15	
BMI (Kg/m^2^)	24.81 ± 2.855 (20.60–30.10)	23.98 ± 4.502 (15.80–33.50)	24.35 ± 4.417 (18.30–34.20)	p = 0.7317
Tobacco	3/10 (F: 1; M: 2)	4/18 (F: 0; M: 4)	4/15 (F: 1; M: 3)	P = 0.896
CD4 Tc^1^ (Cells/mm^3^)	995 (IQR 856.5–1238)	457 (IQR 276–564.5)	559 (IQR 369–703)	HIV SN vs. EFV p = 0.0002 HIV SN vs. PI p = 0.0135 EFV vs. PI p = 0.7453
CD8 Tc^1^ (Cells/mm^3^)	560.5 (IQR 510.8–875.5)	615 (IQR 485.8–762.3)	860 (IQR 634–974)	HIV SN vs. EFV p > 0.99 HIV SN vs. PI p = 0.26 EFV vs. PI p = 0.058
CD4/CD8^1^ ratio	1.55 (IQR1.375–1.85)	0.845 (IQR 0.555-1)	0.67 (IQR 0.47–0.97)	HIV SN vs. EFV p = 0.0011 HIV SN vs. PI p = 0.0002 EFV vs. PI p > 0.99
Viral load (HIV-1 RNA copies/ml)	NA	<40	<40	–
Time on ART (months)	NA	67 ± 30.5	73 ± 33.8	P = 0.875

Body Mass Index (BMI) and age are given as mean ± standard deviation (SD) (minimum-maximum). Time on antiretroviral therapy (ART) data is shown as mean ± standard deviation. ^1^CD4 and CD8 T cell counts and CD4/CD8 ratio are shown as median and interquartile range (IQR). Non-parametric Kruskal-Wallis and two-tailed Fisher´s exact tests were used to compare groups where appropriate using Graph Pad Prism version 6. Abbreviations: HIV SN: HIV seronegative, HIV+ EFV: HIV-infected individuals on Efavirenz-based therapy and HIV+ PI: HIV-infected individuals on ritonavir-booster Protease Inhibitors -based therapy; MSM: men who have sex with men; HTS; heterosexual.

**Table 2:** Comparison of bacterial diversity between HIV SN and HIV+ on EFV and PI-based regimens.

Groups	Chao1	Observed species	PD_whole_tree	Shannon
HIV SN	1174.78 ± 357.48	727.14 ± 228.15	41.16 ± 9.26	5.70 ± 0.77
HIV+ EFV	859.09 ± 254.71	532.61 ± 170.11	30.03 ± 7.66	4.95 ± 0.84
HIV+ PI	797.73 ± 195.90	495.51 ± 123.81	30.52 ± 5.96	4.69 ± 0.73
HIV SN vs HIV+ EFV	0.0225	0.0375	0.0075	0.042
HIV SN vs HIV+ PI	0.012	0.021	0.006	0.018
HIV+ EFV vs HIV+ PI	0.459	0.499	0.848	0.38

Bacterial diversity values are given as mean ± standard deviation at a rarefaction depth of 25,025 sequences per sample and correspond to the first three rows. Alpha diversity was compared between HIV SN and HIV+ EFV and HIV+ PI by means of a non-parametric t-test using the *compare_alpha_diversity.py* script of QIIME. P values were FDR-corrected for multiple comparisons and are shown in the last three rows. Abbreviation: PD_whole_tree: Faith´s phylogenetic diversity, HIV SN: HIV seronegative, HIV+ EFV: HIV-infected individuals on EFV-based and HIV+ PI: HIV-infected individuals on a PI-based regimen.
