# Supplementary material for: Fecal Bacterial Communities in treated HIV infected individuals on two antiretroviral regimens
Source: Sci Rep. 2017 Mar 6;7:43741. doi: 10.1038/srep43741 (PMC5338340; doi:10.1038/srep43741)
Supplement: Supplementary Information [file srep43741-s1.pdf]

Fecal Bacterial Communities in treated HIV infected individuals on two antiretroviral regimens

Sandra Pinto-Cardoso[1], Catherine Lozupone[2], Olivia Briceño[1], Selma Alva-Hernández[1], Norma Téllez[1], Adriana Aguilar[1], Akio Murakami-Ogasawara[1] and Gustavo Reyes-Terán[1*]

Supplemental Information

Supplementary Figures and Tables

Supplementary Figures and Tables are listed in the order they appear in the main document.

Figure S1: EFV- and PI-based ARV regimens display similar levels of bacterial diversity

Figure S2: Taxonomy bar plots showing the bacterial communities in HIV-negative individuals (SN) and HIV-infected individuals on 2 ARV regimens (HIV+ EFV and HIV+ PI) at genus level.

Table S1: Discriminative OTUs by HIV status and ART regimen

Table S2: Levels of immune activation/inflammation in EFV- and PI-based regimens are comparable to HIV SN

Table S3: Significant negative correlations between OTUs from the Clostridiales order and the intestinal barrier dysfunction marker I-FABP

Table S4: Dietary assessment across our study population

Table S5: The relative abundance of Prevotella and Bacteroides and dietary measurements

Table S6: Antibiotic treatment history previous to sample collection

Figure S1: EFV- and PI-based ARV regimens display similar levels of bacterial diversity

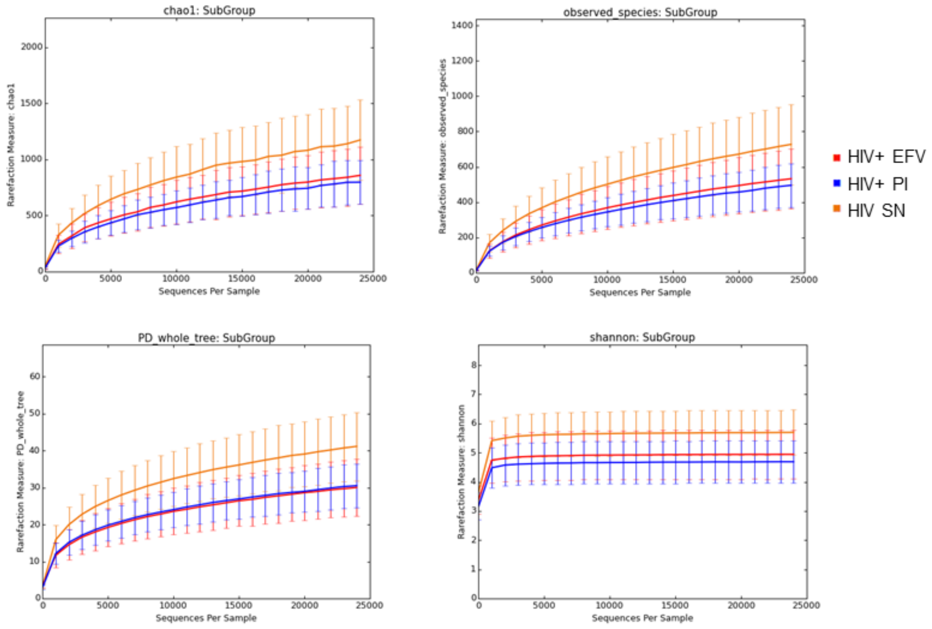

Legend: Rarefaction curves showing ARV regimens impact on bacterial diversity. Four alpha-diversity metrics: Chao1, observed_species, PD_whole_tree (Faith´s Phylogenetic Distance) and shannon; were calculated using *alpha_rarefaction.py* script of QIIME. HIV seronegative individuals (HIV SN), HIV-infected individuals on an EFV-based (HIV+ EFV) and those on a PI-based regimen (HIV+ PI) are shown in orange, red and blue respectively. Rarefaction curves are shown as the average of ten random samplings ± standard deviation at various sampling depths. ARV: antiretroviral.

Supplementary Figure S2: Taxonomy bar plots showing the bacterial communities in HIV-negative individuals (SN) and HIV-infected individuals on 2 ARV regimens (HIV+ EFV and HIV+ PI) at genus level.

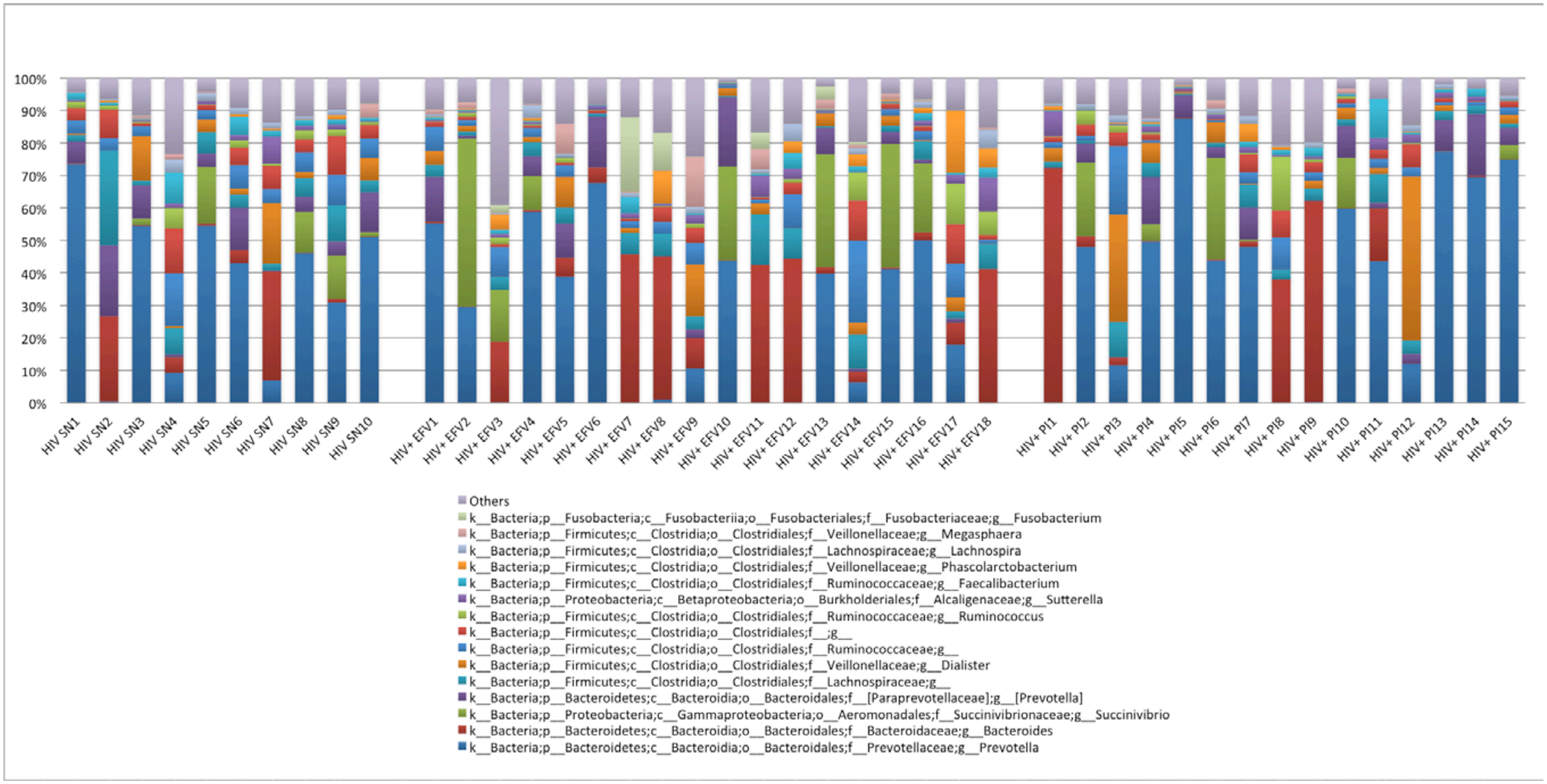

Legend: Stacked taxonomic bar plots showing the relative abundance of the most predominant genera (>1%) in our study cohort. Plots were generated based on the output file otu_table_L6.txt. Taxonomy was assigned using the pick closed OTU strategy in QIIME. Subsequently, the OTU_table.biom was rarified at an even sequencing depth (25,025 sequences per sample). Each bar plot represents an individual in a particular subgroup: HIV SN: HIV seronegative, HIV+ EFV: HIV-infected individuals on EFV-based regimen, and HIV+ PI: HIV-infected individuals on PI-based regimen. Only genera above a relative abundance of 1% are shown. Others represent genera that were present at an overall relative abundance lesser than 1%.

Supplementary Table S1: Discriminative OTUs by HIV status and ART regimen

OTU	P	FDR_P	Bonferroni_P	Mean HIV SN	Mean HIV+ EFV	Mean HIV+ PI	Taxonomy
366068	2.17E-05	0.0084	0.015	55.1	1.22	18.8	f__Ruminococcaceae; g__Faecalibacterium; s__prausnitzii
558599	2.36E-05	0.0084	0.016	11	0.11	0.2	f__Ruminococcaceae; g__; s__
844006	4.98E-05	0.0118	0.035	3	0.44	0.33	f__Ruminococcaceae; g__; s__
173135	0.00021	0.0382	0.153	9.1	4.33	0.46	f__Ruminococcaceae; g__Faecalibacterium; s__prausnitzii

Legend: Discriminant OTUs between HIV seronegative individuals (HIV SN) and HIV+ individuals on Efavirenz (HIV+ EFV) and HIV+ individuals on ritonavir-boosted Protease Inhibitors (PI) were determined by using the group_signifcance.py script of QIIME using nonparametric ANOVA (Kruskal-Wallis). The OTU table was first filtered to remove OTUs that we not present in at least 25 percent. The first column corresponds to the OTU id, the second column to the raw p value, the third and fourth column correspond to the p value after correction for multiple comparisons by Benjamini-Hochberg False Discovery Rate (FDR) and Bonferroni respectively, the fifth, sixth and seventh column represent the mean OTU count for that given category and the last column contains the taxonomic classification. As all discriminant OTUs are from the same taxonomical kingdom (Bacteria), phylum (Firmicutes), class (Clostridia) and order (Clostridiales), and for the purpose of simplifying the table, taxonomic classification was reduced from family to species. Only OTUs that achieved FDP-corrected p values <0.05 are shown.

Supplementary Table S2: ***Levels of immune activation/inflammation in EFV- and PI-based regimens are comparable to HIV SN***

	HIV SN	HIV+ EFV	HIV+ PI	P value
hsCRP (μg/mL)	1.717 ± 1.77<br>(0.589-6.616)	2.755 ± 4.18<br>(0.0406-11.746)	1.721 ± 2.41<br>(0.349-10.036)	0.393
D-Dimer (ng/mL)	1.940 ± 2.9<br>(0.711-10.043)	2.598 ± 1.9<br>(0.820-8.164)	1.945 ± 59.99<br>(0.564-234.209)	0.220
% CD38+HLADR+ CD4+	3.31 ± 1.5<br>(0.63-4.43)	3.235 ± 2.67<br>(0.91-10.3)	2.9 ± 2<br>(0.67-7.49)	0.934
% CD38+HLADR+ CD8+	4.41 ± 2.41<br>(2.03-8.66)	6.27 ± 3.33<br>(1.65-11.4)	5.07 ± 5.95<br>(2-25.9)	0.494

Legend: Markers of inflammation (hs-CRP and D-Dimer) were measured in plasma by ELISA and CD4 and CD8 T cell activation was measured by flow cytometry as the % of cells expressing CD38 and HLA-DR. Comparisons between groups were performed using nonparametric Kruskal-Wallis test. P values shown are adjusted for multiple comparisons. Data is indicated as mean ± standard deviation (minimum-maximum).

Supplementary Table S3: Significant negative correlations between OTUs from the Clostridiales order and the intestinal barrier dysfunction marker I-FABP

OTU ID	Spearman´s Rho	p value	FDR-p value	Taxonomy
529740	-0.586	2.63E-05	0.0124	p__Firmicutes; c__Clostridia; o__Clostridiales; f__Lachnospiraceae; g__; s__
345542	-0.548	0.00011	0.0265	p__Firmicutes; c__Clostridia; o__Clostridiales; f__Lachnospiraceae; g__Roseburia; s__
173135	-0.538	0.00016	0.0265	p__Firmicutes; c__Clostridia; o__Clostridiales; f__Ruminococcaceae; g__Faecalibacterium; s__prausnitzii
367232	-0.530	0.00022	0.0267	p__Firmicutes; c__Clostridia; o__Clostridiales; f__Ruminococcaceae; g__Faecalibacterium; s__prausnitzii
780650	-0.518	0.00033	0.0317	p__Firmicutes; c__Clostridia; o__Clostridiales; f__Clostridiaceae; g__; s__
581003	-0.511	0.00042	0.0334	p__Firmicutes; c__Clostridia; o__Clostridiales; f__; g__; s__
560535	-0.503	0.00054	0.0364	p__Firmicutes; c__Clostridia; o__Clostridiales; f__Ruminococcaceae; g__; s__

Legend: Table showing significant negative correlations between several OTUs from the Clostridiales order and the intestinal barrier dysfunction marker I-FABP. Correlations between the OTU abundances and markers of disease progression (sCD14 for bacterial translocation, I-FABP for intestinal barrier dysfunction, CD38+ HLA-DR+ CD4+ and CD8+ for T cell activation and D-Dimer and hsCRP for residual inflammation) were computed using the *observation_metadata_corrrelation.py* script of QIIME and the Spearman's Rho method. We are only showing the correlations that remained significant after p-values were corrected for multiple comparisons by the Benjamini-Hochberg False Discovery Rate (FDR) method for I-FABP. No correlation was found between individual taxa and sCD14 (FDR $p>0.98$), CD4 T cell activation (FDR $p>0.93$), CD8 T cell activation (FDR $p>0.99$), D-dimer (FDR $p>0.96$) and hsCRP (FDR $p>0.17$) and individual taxa. Abbreviations: OTU: Operational Taxonomic Units, hsCRP: high sensitivity C-reactive protein.

Supplementary Table S4: Dietary assessment across our study population

	HIV SN	HIV+ EFV	HIV+ PI	P-value
**Body Fat Percentage**	30.82 ± 10.04	27.88 ± 8.34	27.39 ± 8.33	p=0.507
**Total Fat Mass (DXA)**	7.7 ± 3.019	6.806 ± 3.287	6.46 ± 2.79	p= 0.454
**Daily Calorie Intake (Kcal)**	2339 ± 590.3	2776 ± 968.6	2609 ± 598.4	p= 0.514
**Protein Intake (g)**	92.62 ± 24.48	106.5 ± 33.8	92.25 ± 23.52	p= 0.489
**Carbohydrate Intake (g)**	287.8 ± 60.91	369.2 ± 136.6	353.4 ± 91.41	p= 0.13
**Fiber Intake (g)**	20.23 ± 4.35	27.07 ± 9.0	23.03 ± 7.43	p= 0.108
**Sugar Intake (g)**	126.9 ± 37.65	123.9 ± 63.67	123.3 ± 48.34	p= 0.901
**Total Fat Intake (g)**	88.52 ± 28.44	100.9 ± 41.84	88.47 ± 24.87	p= 0.7768
**Saturated Fat Intake (g)**	25.22 ± 10.20	28.69 ± 12.05	23.35 ± 8.02	p=0.4358
**BMI (Kg/m^2)**	24.81 ± 2.855	23.98 ± 4.502	24.35 ± 4.417	p= 0.7317

Legend: Dietary intake was assessed by means of a food frequency questionnaire (FFQ) validated for the Mexican population and three 24-hour dietary recalls as described in Methods. Total body composition and fat content was measured via Dual-energy X-ray absorptiometry (DXA). Data is indicated as mean ± standard deviation. Comparisons between groups were performed using nonparametric Kruskal-Wallis test. P values shown are adjusted for multiple comparisons.

Supplementary Table S5: The relative abundance of Prevotella and Bacteroides and dietary measurements

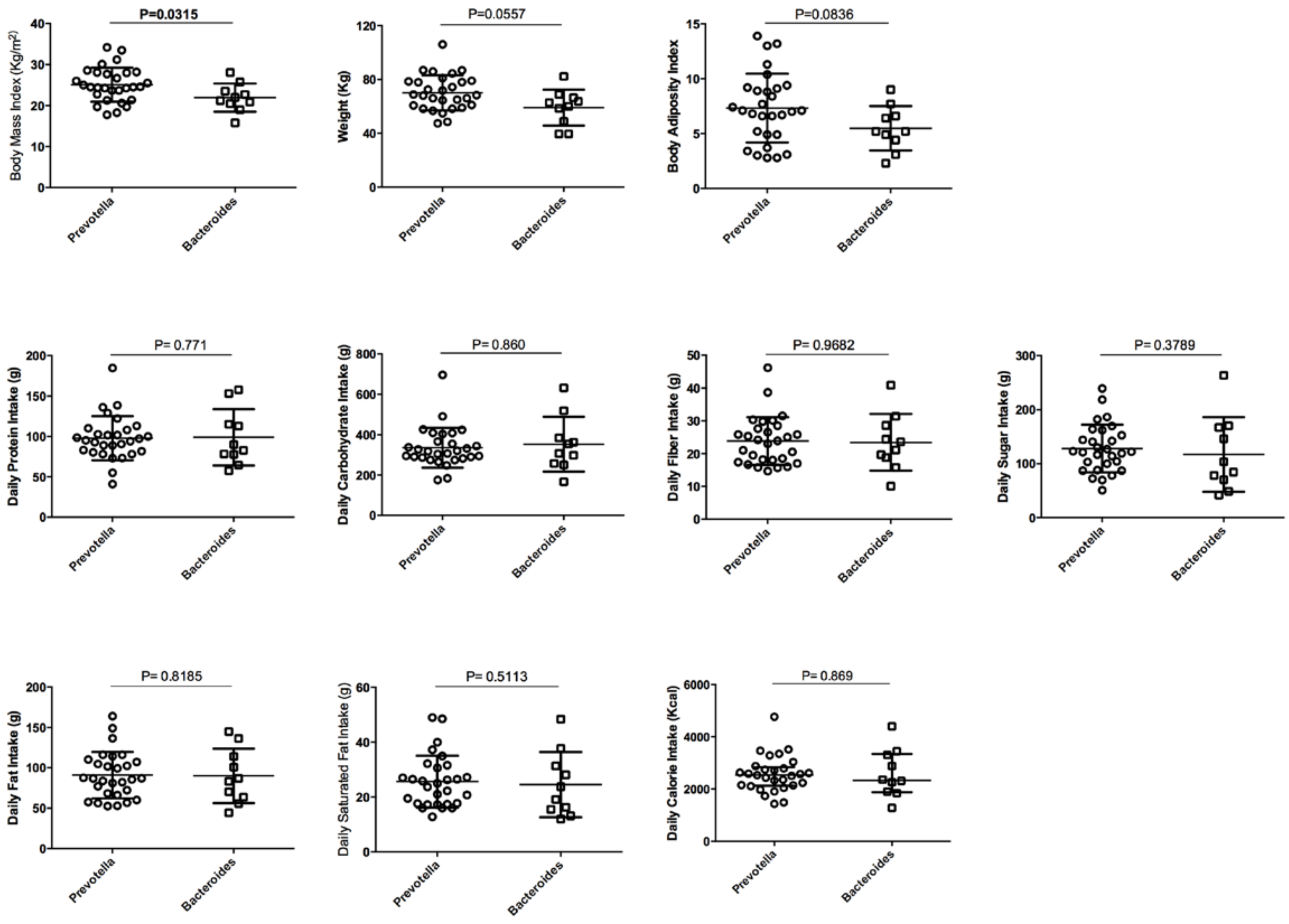

Legend: Scatter plots showing individuals with bacterial communities rich in either Prevotella or Bacteroides in relation to various dietary measurements. Data is plotted as mean ± standard deviation. Line represents the mean. Comparisons between the two groups were performed using Mann´s Whitney U-Test.

Supplementary Table S6: Antibiotic treatment history previous to sample collection

	HIV SN	HIV+ EFV	HIV+ PI	P value
**Time since last Abx (months)**				
**Mean ± SD**	5.778 ± 4.790	7.824 ± 5.175	8.214 ± 3.641	P= 0.3596
**Missing information**	1/10	1/18	1/15	
**Last Antibiotic treatment before sample collection**				
**Ciprofloxacin**	2/9	1/17	2/14	
**Amoxicillin**	2/9	5/17	6/14	
**TMP/SMX**	2/9	2/17	1/14	
**Amoxicillin/clavulin**	1/9	0/17	1/14	
**Penicillin**	0/9	3/17	1/14	
**Terramycin**	0/9	1/17	0/14	
**Levofloxacin**	0/9	0/17	1/14	
**Did not remember**	1/9	6/17	2/14	

Legend: Last antibiotic usage and type of antibiotic taken was recorded at the time of recruitment by the medical staff. Comparisons between groups were performed using nonparametric Kruskal-Wallis test. P values shown are adjusted for multiple comparisons. Data is indicated as mean ± standard deviation. Abbreviations: Abx: antibiotic treatment, HIV SN: HIV seronegative, HIV+ EFV and HIV+ PI: HIV-infected individuals on EFV or PI-based regimens respectively.
